# A comparison of both DENSE and feature tracking techniques with tagging for the cardiovascular magnetic resonance assessment of myocardial strain

**DOI:** 10.1186/s12968-018-0448-9

**Published:** 2018-04-19

**Authors:** J. Jane Cao, Nora Ngai, Lynette Duncanson, Joshua Cheng, Kathleen Gliganic, Qizhi Chen

**Affiliations:** St Francis Hospital, The Heart Center, State University of New York at Stony Brook, Stony Brook, New York USA

**Keywords:** Circumferential strain, Radial strain, Longitudinal strain, Cardiac MRI, Tagging, Displacement encoding with simulated echoes, Feature tracking

## Abstract

**Background:**

Myocardial strain is increasingly recognized as an important assessment for myocardial function. In addition, it also improves outcome prediction. However, there is lack of standardization in strain evaluation by cardiovascular magnetic resonance (CMR). In this study we compared strain values using multiple techniques and multiple vendor products.

**Methods:**

Prospectively recruited patients with cardiomyopathy of diverse etiology (*N* = 77) and healthy controls (*N* = 10) underwent CMR on a 1.5 T scanner. Tagging, displacement encoding with stimulated echoes (DENSE) and balanced stead state free precession cine imaging were acquired on all subjects. A single matched mid left ventricular (LV) short axis plane was used for the comparisons of peak circumferential (Ecc) and radial strain (Err) and a 4-chamber view for longitudinal strain (Ell). Tagging images were analyzed using harmonic phase (HARP) and displacement encoding with stimulated echoes (DENSE) images using a proprietary program. Feature tracking (FT) was evaluated using 3 commercially available software from Tomtec Imaging Systems, Cardiac Image Modeller (CIM), and Circle Cardiovascular Imaging. Tagging data were used as reference. Statistic analyses were performed using paired t-test, intraclass correlation coefficient (ICC), Bland Altman limits of agreement and coefficient of variations.

**Results:**

Average LV ejection fraction was 50% (range 32 to 62%). Regional LV wall motion abnormalities were present in 48% of the analyzed planes. The average Ecc was − 13 ± 4%, − 13 ± 4%, − 16 ± 6%, − 10 ± 3% and − 14 ± 4% for tagging, DENSE, Tomtec, CIM and Circle, respectively, with the best agreement seen in DENSE and Circle with tagging. The Err was highly varied with poor agreement across the techniques, 32 ± 24%, 40 ± 28%, 47 ± 26%, 64 ± 33% and 23 ± 9% for tagging, DENSE, Tomtec, CIM and Circle, respectively. The average Ell was − 14 ± 4%, − 8 ± 3%, − 13 ± 5%, − 11 ± 3% and − 12 ± 4% for tagging, DENSE, Tomtec, CIM and Circle, respectively with the best agreement seen in Tomtec and Circle with tagging. In the intra- and inter-observer agreement analysis the reproducibility of each technique was good except for Err by HARP.

**Conclusions:**

Small but important differences are evident in Ecc and Ell comparisons among vendors while large differences are seen in Err assessment. Our findings suggest that CMR strain values are technique and vendor dependent. Hence, it is essential to develop reference standard from each technique and analytical product for clinical use, and to sequentially compare patient data using the same software.

**Electronic supplementary material:**

The online version of this article (10.1186/s12968-018-0448-9) contains supplementary material, which is available to authorized users.

## Background

Myocardial strain is increasingly recognized as an important myocardial performance index [1, 2]. It can detect the decline of myocardial function preceding the reduction of left ventricular (LV) ejection fraction [3]. A growing body of literature largely from echocardiography has demonstrated that reduced longitudinal strain is associated with adverse clinical outcome with and without reduced ejection fraction [4]. As a result, the assessment of longitudinal strain is now recommended in the ACC/AHA guideline to be routinely performed in clinical echocardiographic evaluation [5].

Strain can be analyzed using a number of techniques by cardiovascular magnetic resonance (CMR) [6]. Tagged myocardial deformation is the first noninvasive technique that was successfully used in strain evaluation [7–9]. By adding grids or lines to the imaging plane myocardial deformation can be quantitatively analyzed. Displacement encoding with stimulated echoes (DENSE) is a technique that encodes the tissue displacement directly into the phase of the CMR signal. The development of DENSE has contributed to improved spatial resolution compared with harmonic phase magnetic resonance (HARP) tagging in assessing myocardial deformation, dependent on the acquisition matrix and the size of the filter used [10, 11]. More recently, feature tracking (FT) of the cine images has provided a new dimension for strain analysis by directly tracking the features on routine CMR cine images [12–14]. While each technique has its merit, the difference of physics in image acquisition and of algorithm in post processing may inevitably contribute to differences in strain values. In this study we sought to evaluate 3 techniques including tagging, DENSE and FT for LV strain evaluation. In addition, we compared three commercially available FT programs. HARP tagging was chosen as the reference because this is the most commonly used reference technique in the literature for strain comparison.

## Methods

The study protocol was approved by the Institutional Review Board. All participants were recruited prospectively after obtaining written informed consent. Healthy subjects were recruited if they did not have cardiovascular history or risk factors and had normal electrocardiogram (ECG) and transthoracic echocardiogram. Patients with arrhythmia at the time of CMR were excluded from the analysis. Additional exclusion criteria were any contraindication to CMR (claustrophobia, CMR incompatible metals), allergic reaction to gadolinium based contrast agent or impaired renal function (estimated glomerular filtration rate < 45 mL/min/1.7m^2^). All study participants completed a questionnaire for demographic information and medical history.

### CMR image acquisition

All subjects underwent CMR imaging in a 1.5 T Avanto scanner (Siemens Healthineers Erlangen, Germany). A combination of 16-elements phased array surface coil and spine coil were used. LV volumes and systolic function were assessed using breath-hold balanced steady state free precession cine images with retrospective ECG gating. A stack of LV short axis planes and three long axis planes (2-, 3-, and 4-chamber) were obtained by using the following imaging parameters: field-of-view (FOV) = 240 × 240 mm^2^ to 260 × 260 mm^2^, echo spacing is 3.15 ms, repetition time (TR) 48 ms to 84 ms, average temporal resolution 50 ms, flip angle 58° to 70°, image matrix 192 × 192 for long axis and 154 × 192 for short axis planes, average voxel size 1.3 × 1.3 × 6 mm^3^ for long axis and 1.6 × 1.3 × 8 mm for short axis views. Image was reconstructed with interpolation. The average breath hold was ~ 12 s.

Complementary spatial modulation of magnetization (CSPAMM) tagged imaging used a spoiled gradient echo grid-tag cine sequence and retrospective ECG gating. The retrospective ECG gating was chosen because the reconstructed images began close to 0 ms for the first frame matching that of feature tracking as opposed to 20 to 25 ms offset by the prospective gating. The typical sequence parameters were as follows: FOV 270 × 360 mm^2^, TR 78.0 ms, (echo time) TE 4.0 ms, grid tag spacing 8 mm, flip angle 12°, echo spacing 8.8 ms, with voxel size 1.4 × 1.4 × 6 mm^3^. Image was reconstructed with interpolation. The average breath hold was ~ 18 s.

A CSPAMM spiral cine DENSE pulse sequence with prospective ECG gating was used to acquire DENSE images with displacement encoding applied in two orthogonal in-plane directions. A combined acquisition of both displacement encoding directions was obtained. The imaging parameters included FOV = 360 × 360 mm^2^, flip angle = 20°, TR = 15 ms, TE = 1 ms, number of spiral interleaves = 6, temporal resolution = 30 ms, displacement encoding frequency = 0.1 cycles/mm and voxel size 2.8 × 2.8 × 8 mm^3^. The average breath hold was ~ 23 s. On average, 80% of the cardiac cycle was captured.

For late gadolinium enhancement (LGE) imaging a phase sensitive inversion recovery (PSIR) gradient echo sequence was performed 8-10 min after the administration 0.15 mmol/kg of gadopentetate dimenglumine (Magnevist, Bayer Healthcare Pharmaceuticals, Berlin, Germany). The typical sequence parameters included FOV 360 × 290 mm^2^, TE 3.17 ms, TR = 1 × RR interval, flip angle 25°, voxel size 1.9 × 1.4 × 8 mm^3^. An inversion time (TI) scout with increasing TI value was performed on mid ventricular short axis slice to determine the TI that allowed the optimal nulling of normal myocardium before the LGE images were obtained in a stack of LV short axis slices and in 2-, 3- and 4-chamber views.

### Image analysis

Volumetric analysis for quantification of LV volumes and ejection fraction was obtained on short axis cine images using commercially available QMASS software (Medis BV, Leiden, Netherlands). LV volume and mass were normalized to body surface area. Left atrial (LA) volumes were calculated using a biplane area and length method following the formula: 0.85xA1xA2/L, where A1 and A2 were areas measured by planimetry in 2- and 4-chamber views, respectively and L was the length of LA perpendicular to the center of mitral annulus in the 4-chamber plane [15]. The circumferential (Ecc) and radial strain (Err) were analyzed in one mid-ventricular short axis plane. The slice position was carefully matched for all techniques. Lagrangian strain was used by each of the techniques. The longitudinal strain (Ell) was analyzed in 4-chamber view.

Tagged images were analyzed using HARP software (Myocardial Solutions, Morrisville North Carolina, USA). LV endocardial and epicardial contours were manually drawn in the first phase, which was then automatically propagated. The degradation of tags was common in diastole. Depending on heart rate and image quality 15 up to 20 phases were analyzable. Manual adjustment of contours was provided when automatic tracking failed. On average, it took at least 15 min for an experienced analyst to process one slice.

Software for DENSE analysis was proprietary (University of Auckland, New Zealand). The myocardium was defined by placing the guide points on endo- and epicardium in addition to the center of LV cavity and the right ventricular insertion points. Contours were drawn in diastole and automatically propagated to all cardiac phases. Post processing took 2-3 min per slice.

FT analyses were performed using the following programs, Tomtec (Image Arena, Version 4.6, Tomtec Imaging Systems, Unterschleissheim, Germany), CIM (Cardiac Image Modeller, Version 8.1.5, The University of Auckland, Auckland, New Zealand) and Circle (CMR42, version 5.6.5, Cardiovascular Imaging Inc., Calgary, Alberta, Canada). LV contours were obtained by manually tracing the LV endocardium and epicardium at end diastole followed by automatic propagating contours to all phases [16]. Contours were manually corrected when automatic tracking failed. Analysis took 1–3 min per slice for an experienced analyst.

**Fig. 1:**
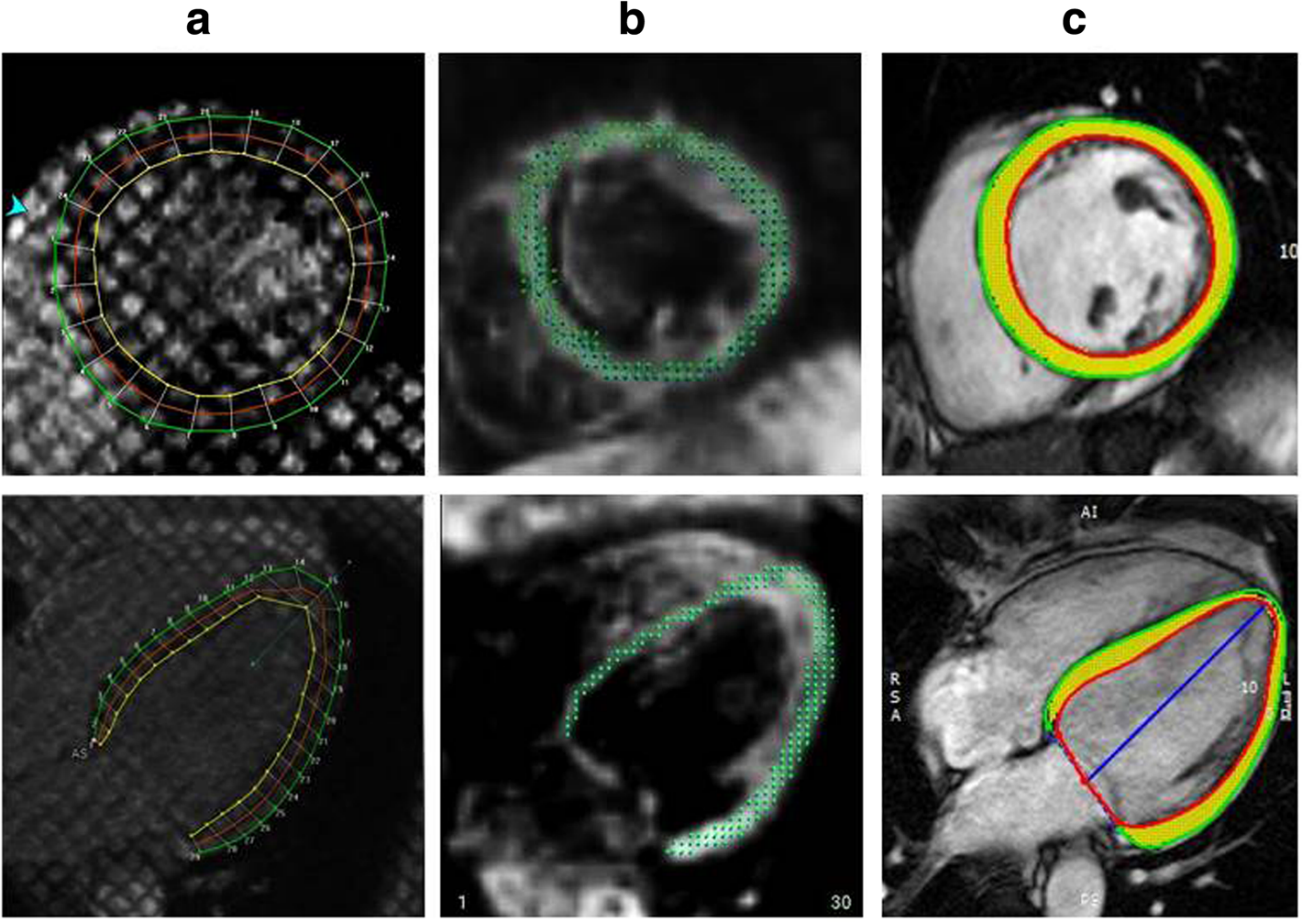
Examples of images by tagging (**a**), displacement encoding with stimulated echoes (DENSE) (**b**) and feature tracking (**c**) analysis with corresponding contour overlay in left ventricular short axis and 4-chamber views. In tagging, the endocardial and epicardial borders are marked by yellow and green contours, respectively. In DENSE, the images are shown in diastole with points on myocardium depicting the 2D displacement field. In feature tracking, the endocardial and epicardial borders are marked by red and green contours, respectively with myocardium marked in yellow

**Fig. 2:**
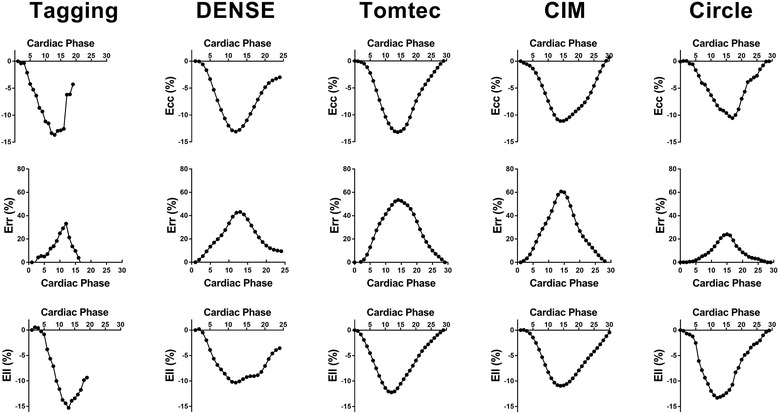
Examples of strain curves from circumferential (Ecc), radial (Err) and longitudinal strain (Ell) from the same patient using tagging, displacement encoding with simulated echoes (DENSE) and three feature tracking programs; The average cardiac cycle length is 844 ms. There are fewer phases in tagging and in DENSE imaging due to degradation of tags in diastole and incomplete diastolic acquisition from prospective ECG gating, respectively

Examples of contours from tagging, DENSE and FT are shown in Fig. 1. The corresponding videos are available online Additional files 1, 2, 3, 4, 5 and 6. The typical strain output from tagging, DENSE and feature tracking is illustrated in Fig. 2 using five techniques from the same patient. The tagging strain curves were not always smooth. Although analyzing fitted curve was an option in HARP, we chose to analyze the raw data points as shown in Fig. 2.

Intra- and inter-observer variability was analyzed in ten randomly selected cases for each strain type using all five techniques.

### Statistic analysis

Continuous variables were presented as mean ± standard deviation (SD) and categorical variables as frequencies and proportions. Paired t-test, intraclass correlation coefficient (ICC) and Bland Altman limits of agreement were used for strain comparisons across techniques. ICC and coefficient of variations were used for inter- and intra-observer agreement analysis. All *p*-values were considered to be significant when < 0.05. The analysis was performed using SPSS Statistic software for Windows, Version 22.0 (International Business Machines, Inc., Armonk, New York, USA).

## Results

There were 95 subjects recruited. All had acceptable image quality for FT analysis. However, the suboptimal quality was seen in tagging (*N* = 8) and DENSE (*N* = 3) images. The possible causes included poor breath-hold, body motion and arrhythmia. Therefore, the final analysis cohort consisted of 87 subjects with ten healthy controls and 77 patients with a cardiomyopathy of diverse etiology (Table 1). Mean LV ejection fraction was 50% ranging from 32 to 62%. An infarct pattern LGE was found in 19% (*n* = 17) and 29% (*n* = 27) had a non-infarct pattern. Regional LV wall motion abnormalities were present in 48% (*N* = 52) of the images analyzed including those due to left bundle branch block.

**Table 1:** Participants’ characteristics

Variables	*N* = 87 Mean ± SD or N(%)
Age	58 ± 13
Male	65(71)
Body mass index (Kg/m^2^)	29 ± 5
Heart rate (bpm)	69 ± 11
Systolic blood pressure (mmHg)	129 ± 20
Diastolic blood pressure (mmHg)	74 ± 12
Left bundle branch block on ECG	6(7)
Clinical history	
Hypertension	44(48)
Hyperlipidemia	52(57)
Diabetes mellitus	17(18)
Ever smoked	33(36)
Heart failure	17(18)
Myocardial infarction	9(10)
Coronary stent	27(32)
Coronary bypass graft	13(14)
CMR assessment	
Left ventricular diastolic volume (ml/m^2^)	86 ± 29
Left ventricular systolic volume (ml/m^2^)	46 ± 27
Left ventricular ejection fraction (%)	50 ± 12
Left ventricular mass (g/m^2^)	62 ± 16
Regional wall motion abnormality in the analyzed images	48 (52)
Left atrial volume (ml/m^2^)	39 ± 16
Late gadolinium enhancement	45 (49)
Infarct pattern	17 (19)
Non-infarct pattern	27 (29)
CMR diagnosis	
Left ventricular hypertrophy	13(14)
Myocardial infarction	17(18)
Sarcoidosis	9(10)
Myocarditis	5(5)
Dilated cardiomyopathy	6(7)
Hypertrophic cardiomyopathy	2(3)
Amyloidosis	1(1)
Churg-strauss endomyocarditis	1(1)

Representative strain curves are shown in Fig. 2, where peak strain was assessed using all 5 techniques from the same patient. There were fewer phases of tagging images due to degradation of tags in diastole. Similarly, there were fewer phases in DENSE as a result of prospectively gated imaging acquisition. The peak Ecc was comparable across the techniques with the highest strain value by Tomtec. The peak Ell was also comparable with the exception of significantly lower values by DENSE. In contrast, the peak Err values differed substantially and lacked agreement amongst the techniques.

**Fig. 3:**
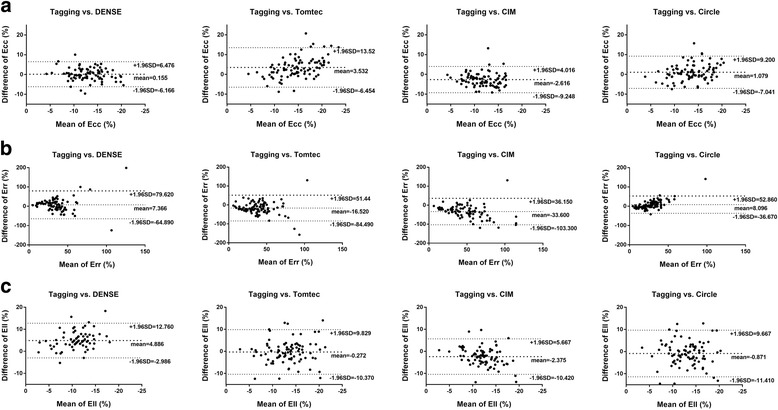
Bland Altman plots comparing DENSE and feature tracking with tagging on the analysis of circumferential (Ecc) (**a**), radial (Err) (**b**) and longitudinal strains (Ell) (**c**) with the y-axis showing the strain differences subtracting strain by the comparing technique from strain by tagging

The average strain value for the normal controls and for the whole cohort is shown in Table 2. In the whole cohort analysis, DENSE showed the best agreement with tagging in Ecc comparison with ICC 0.778 and mean bias of 0.155% in Bland Altman limits of agreement analysis. This was followed by Circle with ICC 0.652 and mean bias of 1.079% (Table 3). Overall the Ecc difference was relatively small across all techniques (Fig. 3a). When compared with tagging there was a small overestimation using all methods except CIM. In contrast, Err values highly varied from technique to technique (Table 2). The agreement was poor with tagging from all techniques (Table 3) as shown in the plots of Bland Altman limits of agreement (Fig. 3b) due largely to overestimation except Circle. In Ell assessment, FT by Tomtec and Circle had the best agreement with tagging rendering a mean bias less than 1.0% in both comparisons. Ell by DENSE, on the other hand, had a large mean bias of 4.9% due to underestimation when compared to tagging (Fig. 3c).

**Table 2:** Comparisons of peak strain assessed by different CMR techniques

	Tagging by HARP	DENSE	FT by Tomtec	FT By CIM	FT By Circle
Entire cohort (*N* = 87)					
Ecc (%)	−13 ± 4	−13 ± 4	−16 ± 6	− 10 ± 3	−14 ± 4
Err (%)	32 ± 24	40 ± 28	47 ± 26	64 ± 33	23 ± 9
Ell (%)	−14 ± 4	−8 ± 3	−13 ± 5	−11 ± 3	−12 ± 4
Normal subjects (*N* = 10)					
Ecc (%)	−15 ± 2	−17 ± 4	−20 ± 4	−12 ± 3	−17 ± 3
Err (%)	38 ± 20	47 ± 14	54 ± 20	76 ± 35	27 ± 6
Ell (%)	−16 ± 3	−11 ± 3	−14 ± 3	−13 ± 2	−14 ± 4

Abbreviation: *Ecc* circumferential strain, *Err* radial strain, *Ell* longitudinal strain

**Table 3:** Comparisons of DENSE and feature tracking with HARP tagging in peak strain assessment

Software	Peak strain	*P* value ^a^	ICC	Mean difference and 95% CI by Bland-Altman
DENSE	Ecc	0.663	0.778	0.155 (−6.166, 6.476)
	Err	0.072	0.032	7.366 (−64.89, 79.62)
	Ell	< 0.001	0.359	4.886 (−2.986, 12.76)
Tomtec	Ecc	< 0.001	0.507	3.532 (− 6.454, 13.52)
	Err	< 0.001	0.095	−16.52 (−84.49, 51.44)
	Ell	0.290	0.587	−0.2719 (−10.37, 9.829)
CIM	Ecc	< 0.001	0.571	−2.616 (−9.248, 4.016)
	Err	< 0.001	0.284	−33.6 (− 103.3, 36.15)
	Ell	< 0.001	0.527	−2.375 (− 10.42, 5.667)
Circle	Ecc	0.036	0.652	1.079 (−7.041, 9.2)
	Err	< 0.001	0.312	8.096 (−36.67, 52.86)
	Ell	0.049	0.408	−0.8713 (−11.41, 9.667)

Abbreviations: *Ecc* circumferential strain, *Err* radial strain, *Ell* longitudinal strain, *ICC* intraclass correlation coefficient, *CI* confidence interval

^a^Paired t-test

In the reproducibility analysis, the intra-observer agreement was good for all strain analyses (Table 4). While the inter-observer agreement was good for all Ecc evaluation, the agreement on Ell and Err evaluation was less consistent. We also analyzed coefficient of variations of the intra- and inter-observer agreement (Table 5). Of all the techniques, HARP had the largest data dispersion shown by the highest coefficient of variations in all strain analyses. In contrast, DENSE had small data dispersion with the exception of Err from inter-observer comparison. Overall, FT showed relatively small data dispersion for all comparisons consistently.

**Table 4:** Inter- and intra-observer variability by intraclass correlation coefficient

Technique	Intra-observer		Inter-observer	
	ICC	*p* value	ICC	*p* value
Tagging				
Ecc	0.982	< 0.001	0.909	< 0.001
Err	0.936	< 0.001	0.307	0.277
Ell	0.835	0.002	0.700	0.029
DENSE				
Ecc	0.961	< 0.001	0.973	< 0.001
Err	0.948	< 0.001	0.644	0.027
Ell	0.761	0.004	0.476	0.122
FT-Tomtec				
Ecc	0.993	< 0.001	0.992	< 0.001
Err	0.950	< 0.001	0.993	0.000
Ell	0.938	< 0.001	0.921	< 0.001
FT-CIM				
Ecc	0.935	< 0.001	0.955	< 0.001
Err	0.864	< 0.001	0.963	< 0.001
Ell	0.944	< 0.001	0.987	0.000
FT-Circle				
Ecc	0.947	< 0.001	0.865	0.002
Err	0.849	0.003	0.762	0.018
Ell	0.910	< 0.001	0.948	< 0.001

Abbreviations: *Ecc* circumferential strain, *Err* radial strain, *Ell* longitudinal strain, *ICC* intraclass correlation coefficient

**Table 5:** Coefficient of variations of the Inter- and intra-observer agreement

	Intra-observer (%)	Inter-observer (%)
Ecc		
HARP	4.4	8.5
DENSE	0.2	0.9
CIM	1.8	0.5
Tomtec	1.4	2.9
Circle	0.4	0.3
Err		
HARP	14.4	129.2
DENSE	1.4	50.5
CIM	4.4	1.9
Tomtec	2.6	< 0.1
Circle	4.4	4.3
Ell		
HARP	6.4	31.5
DENSE	2.0	0.2
CIM	0.5	0.7
Tomtec	3.4	1.1
Circle	0.4	0.3

Abbreviations: *Ecc* circumferential strain, *Err* radial strain, *Ell* longitudinal strain

## Discussion

In this study we observed good agreement in most of the comparisons for Ecc across all techniques with DENSE and FT with Circle software having the best agreement with tagging. Ecc by Tomtec software appeared to have higher values resulting in modest agreement with tagging. Unlike Ecc, Err agreement was poor across all techniques. In the Ell comparisons, Tomtec and Circle had good agreement with tagging. Ell by DENSE appeared to be consistently lower than Ell by other techniques. Overall, reproducibility was good for all techniques studied especially in the intra-observer agreement despite the differences present between techniques.

Tagged imaging is widely available and frequently used as the reference standard for CMR FT and for speckle tracking in echocardiography [17, 18]. The broad use of tagging should be partly credited to HARP, a commercially available program allowing for fast tagging analysis. DENSE, on the other hand, remains largely as a research tool since the sequence is not yet commercialized [11, 19]. DENSE encodes tissue displacement directly into the phase of the CMR signal [20]. When the two orthogonal displacement encodings are acquired in separate breath-hold the temporal resolution of DENSE can be as high as 17 ms [11]. However, this approach demands perfect breath-hold, exact body position and matched heart rate between the two acquisitions, which can be challenging in patients with advanced morbidity. This is why a single breath-hold approach was used in our study for combined image acquisition in orthogonal directions of displacement encoding trading reduced temporal resolution for consistent image quality. Both DENSE and tagging provide encoding to the myocardium thereby allowing direct tracking of the myocardial deformation. However, the dedicated imaging can potentially be a burden to patients. Arrhythmia, motion and breath-hold difficulty can lead to inadequate image quality. In this clinical cohort, we observed poor image quality in three DENSE and eight tagged images causing considerable data loss. In contrast, FT uses images from routine cine imaging that is typically acquired with shorter acquisition time than DENSE or tagging and therefore is usually better tolerated by patients. Using the arrhythmia rejection protocol balanced steady state free precession cine imaging can produce good image quality even with arrhythmia. That is why we observed 100% usable images. It should be noted that there are diverse algorithms within FT technology which may in part contribute to the differences of strain value as some algorithms track the endocardial border while others track the full myocardial property. For example, Tomtec software tracks the endocardial border thereby yielding higher values than those of other FT products. The spatial resolution of each technique can also vary. For example, the gradient echo for tagged imaging has an in-plane resolution of 1.4 × 1.4 mm. However, it is reduced when analyzed by HARP due to signal filtering. In the case of feature tracking, while the spatial resolution of cine image is maintained, the true spatial resolution is difficult to determine because the exact spacing of the features, likely different from software to software, is usually a proprietary knowledge and unknown to the user.

There are increasing publications that include CMR strain evaluation for a variety of clinical conditions including ischemic heart disease [21, 22], hypertrophic cardiomyopathy [23], amyloidosis [24, 25] and cardiomyopathy due to Duchene’s muscular dystrophy [26]. Limited but emerging data support the prognostic value of longitudinal strain by FT [27]. Normal strain value has also been published for reference purpose. Meanwhile, data from a small study shows that strain value by FT differs between vendors [28] thereby raising concerns for mixed use of different FT programs in clinical evaluation. To date, few studies have reported comprehensive comparisons of strain analysis that include tagging, DENSE, and FT. In this report we demonstrate that there is an important difference in strain value among different techniques and FT software products despite good reproducibility of each product. Therefore, we believe the difference in strain value is likely the result of systemic difference in technology whether at the level of image acquisition or at the level of image post processing. Despite multiple commercially available products the design of the FT algorithm remains unknown and largely proprietary, which makes it difficult to fully understand the difference observed in our study.

The variability of CMR derived strain value demonstrated by us and by others suggests that it is necessary to standardize strain evaluation in order to implement clinical strain assessment and to maintain the same software analysis in comparing serial patient data. Before a standardized approach is established it is essential to create reference value not only based on technique but also by vendor specific product.

Our study has several limitations. We chose one product sequence and the commercially available software for tagging analysis. It is plausible that other sequences and or analysis techniques may yield different strain findings. The same drawback may also be true for DENSE imaging as there are many other options available in imaging and in post processing. Similarly, there are growing numbers of vendors that offer FT analysis. It is beyond the scope of this study to include all of them or their various versions. Nonetheless, our findings provide important insight that the strain value is technique and vendor dependent. The choice of tagging by HARP as the reference standard was an empirical decision based on its broad use, which can be challenged especially in the case of Err and Ell evaluation, given their modest reproducibility. It should also be noted that the tags were largely analyzable during systole and subject to fading during diastole. As a result, the number of cardiac phases available for analysis varied from case to case. While in theory the same effect of signal fading is expected from DENSE we found DENSE consistently provided more analyzable phases than tagging. We also recognize that this is a single center experience. However, we believe that our findings can be generalized because our cohort size is relatively large and we have included diverse types of cardiomyopathy representing a typical clinical cohort for CMR evaluation.

## Conclusions

Small but important differences are evident in Ecc and Ell assessment across techniques in addition to large differences seen in Err evaluations. Our findings suggest that it is essential to develop reference standard for each technique or software product for strain evaluation and that analysis software should be maintained consistent for serial patient evaluations. Future research should investigate standardization of strain by CMR.

## Additional files

Additional file 1: **Movie S1.** tagged imaging of short axis view. (AVI 2129 kb)

Additional file 2: **Movie S2.** tagged imaging of 4-chamber view. (AVI 1414 kb)

Additional file 3: **Movie S3.** DENSE imaging of short axis view with strain vector overlay. (AVI 11479 kb)

Additional file 4: **Movie S4.** DENSE imaging of 4-chamber view with strain vector overlay. (AVI 11479 kb)

Additional file 5: **Movie S5.** bSSFP cine imaging of short axis view with feature tracking contours. (MPG 13596 kb)

Additional file 6: **Movie S6.** bSSFP cine imaging of 4-chamber view with feature tracking contours. (MPG 13596 kb)

## Abbreviations

CIM: Cardiac image modeller
CMR: Cardiovascular magnetic resonance
CSPAMM: Complementary spatial modulation of magnetization
DENSE: Displacement encoding with stimulated echoes
Ecc: Peak circumferential strain
Ell: Peak longitudinal strain
Err: Peak radial strain
FOV: Field-of-view
FT: Feature tracking
HARP: Harmonic phase
ICC: Intraclass correlation coefficient
LGE: Late gadolinium enhancement
LV: Left ventricle/left ventricular
PSIR: Phase sensitive inversion recovery
SD: Standard deviation
TE: Echo time
TI: Inversion time
TR: Repetition time
